# Constitutive Cleavage of the Single-Pass Transmembrane Protein Alcadeinα Prevents Aberrant Peripheral Retention of Kinesin-1

**DOI:** 10.1371/journal.pone.0043058

**Published:** 2012-08-08

**Authors:** Chiaki Maruta, Yuhki Saito, Saori Hata, Naoya Gotoh, Toshiharu Suzuki, Tohru Yamamoto

**Affiliations:** Laboratory of Neuroscience, Faculty of Pharmaceutical Sciences, Hokkaido University, Sapporo, Japan; Thomas Jefferson University, United States of America

## Abstract

Various membrane proteins are shed by proteinases, constitutively and/or when stimulated by external signals. While the physiological significance of external signal-induced cleavages has been intensely investigated, relatively little is known about the function of constitutive cleavages. Alcadeinα (Alcα; also called Calsyntenin-1) is an evolutionarily conserved type I single-pass transmembrane protein that binds to kinesin-1 light chain (KLC) to activate kinesin-1's transport of Alcα-containing vesicles. We found that Alcα was constitutively and efficiently cleaved to liberate its ectodomain into the extracellular space, and that full-length Alcα protein was rarely detected on the cell surface. The secretion efficiency of the ectodomain was unaltered by a mutation that both abolished Alcα's KLC-binding activity and attenuated its peripheral transport, suggesting that Alcα's cleavage occurred, at least partly, en route to the cell surface. We further demonstrated that uncleavable mutant Alcα proteins readily accumulated on the cell surface and induced aberrant peripheral recruitment of KLC1 and kinesin heavy chain. Our observations suggest that Alcα is efficiently processed in part to minimize the inappropriate peripheral retention of kinesin-1. This role might exemplify the functional relevance of the constitutive cleavage of single-pass transmembrane proteins.

## Introduction

The cleavage of a membrane protein can be a major regulating event in the activation, modification, or elimination of its function. A wide variety of membrane proteins are proteolyzed constitutively and/or in response to external signals. The molecular mechanisms and physiological significance of external signal-induced cleavage have been intensely investigated. For example, Notch is cleaved by ADAM10 upon binding to its ligand Delta, whose endocytosis mechanically induces a conformational change of the bound Notch receptor to expose its cleavage site (reviewed in [1]). The availability of EGF family growth factors produced as type I transmembrane proteins is regulated by extracellular proteases that cleave them to release soluble ligands, in response to external stimuli, such as a wound (reviewed in [2]). The ectodomain of CD44, the major cell-surface receptor for hyaluronan, is shed following cleavage by membrane-associated metalloproteases, including MMP14, ADAM10, and ADAM17, to regulate cell motility (reviewed in [3]). However, relatively little is known about the functions of the constitutive cleavage of type I transmembrane proteins.

Alcadeinα (Alcα; also called Calsyntenin-1) is an evolutionarily conserved single-pass type I transmembrane protein that is primarily expressed by neurons [4], [5]. Alcα has two splice variants, Alcα1 and Alcα2; Alcα1 is the major product of the gene, and Alcα2 contains 10 extra amino acids in the N-terminal extracellular region. Hereinafter, we refer to Alcα1 simply as “Alcα” unless stated otherwise. Alcα interacts with a light chain subunit of kinesin-1 (KLC) through its small (∼10 amino acids) WD motif, to activate kinesin-1's association with Alcα–containing vesicles and their anterograde transport [6], [7], [8]. The Alcα protein is constitutively cleaved by ADAM10 or ADAM17 to produce the N-terminal extracellular ectodomain (sAlcα) and residual C-terminal fragment (Alcα CTF) [9]. Alcα CTF is subsequently digested by γ-secretase to produce the N-terminal p3-Alcα peptide and the C-terminal intracellular cytoplasmic domain (Alcα ICD) [10]. However, the functional relevance of the constitutive cleavage of Alcα remains elusive, and it is unclear where and how much of the Alcα protein is cleaved in cells.

To examine the functional relevance of the constitutive cleavage of Alcα, we quantitatively analyzed the amount of ectodomain released extracellularly. We found that Alcα was efficiently cleaved to liberate the ectodomain, and its production rate was unexpectedly high: as much as ∼20% of the release rate of a freely secreted protein used as a standard. We also found that the full-length Alcα protein was rarely detected on the cell surface, whereas Alcα was indeed transported to the plasma membrane, suggesting that Alcα was cleaved en route to and/or upon reaching the plasma membrane. We further showed that an ‘uncleavable’ mutant Alcα (with a mutation in the cleavage site) readily accumulated on the cell surface, and caused the aberrant peripheral retention of kinesin-1. These findings suggested that the efficient constitutive cleavage of Alcα partly acts to prevent the inappropriate peripheral retention of kinesin-1, which could impair intracellular trafficking.

## Materials and Methods

### Plasmids

The construction of pcDNA3.1-hAlcα1, pcDNA3.1-FLAG-hAlcα1, pcDNA3.1-Alcα1-FLAG, pcDNA3.1-FLAG-hAPP, pcDNA3.1-hAPP-FLAG, pEGFP-KIF5C, and pcDNA3.1-FLAG-KLC1 was previously described [7]. Plasmids expressing point mutations of Alcα1 resulting in a tryptophan-to-alanine substitution (Alcα WA mutants) were prepared by replacing the target sequence with the corresponding sequence carrying the indicated mutation, generated by PCR. Plasmids expressing N-terminal human placental alkaline phosphatase (AP) fusion proteins were generated by inserting the indicated PCR-amplified fragments into the *Xho*I (AP-Alcα1, AP-APP) or *Xba*I (AP-ephrinB1) site of AP-tag5 [11]. Substitution mutants of the Alcα primary cleavage site (pcDNA3.1-FLAGmt-hAlcα1, pcDNA3.1-FLAGmt-FLAG-hAlcα1, pcDNA3.1-HAmt-hAlcα1, and pcDNA3.1-HAmt-FLAG-hAlcα1) were generated by the megaprimer PCR method [12]. Plasmids expressing primary cleavage site substitution mutants or the AP-fusion form of Alcα with WA mutations (pcDNA3.1-FLAGmt-hAlcα1WA, pcDNA3.1-FLAGmt-FLAG-hAlcα1WA, pcDNA3.1-HAmt-hAlcα1WA, pcDNA3.1-HAmt-FLAG-hAlcα1WA, and AP-Alcα1WA) were prepared by exchanging the C-terminal region of each Alcα to that of the WA mutant. The resultant plasmids were verified by sequencing.

### Antibodies and western blotting

The following antibodies were used. Anti-FLAG mouse monoclonal antibody (M2, Sigma-Aldrich; St. Louis, MO), anti-α-tubulin mouse monoclonal antibody (DM1A, Santa Cruz Biotechnology; Delaware, CA), rabbit anti-GFP (598, Medical & Biological Laboratories; Nagoya, Japan), anti-APP (APP/C, Sigma-Aldrich), guinea pig anti-Alcα (col90; [7]), and rabbit anti-Alcα (UT83; [7]). Western blotting was performed as described [13].

### Cell culture, transfection with plasmids, and immunocytochemistry

CAD mouse neuroblastoma cells were cultured and transfected with expression plasmids as described previously [7]. For immunocytochemistry, expression plasmids were transferred into CAD cells cultured on poly-D-lysine-coated 8-well cover-glass chamber slides (Nalge Nunc; Rochester, NY) with LipofectAMINE 2000 (Invitrogen; Carlsbad, CA). The primary culture of mouse cortical neurons was carried out as described [13]. Immunocytochemistry was performed as described [7]. The bound primary antibodies were visualized with FITC-, Cy3-, or Cy5-conjugated donkey anti-mouse, -rabbit, or -guinea pig IgG, each of which showed minimum cross-reaction with the serum proteins of other species (Jackson ImmunoResearch Laboratories; West Grove, PA).

### Cell surface biotinylation assay and live cell staining

Cell surface biotinylation assay was carried out as described [14]. Briefly, CAD cells or mouse embryonic cortical neurons cultured on 12-well plates (Corning, New York, NY) were washed with ice-cold PBS, then incubated with 0.5 mg/ml EZ-Link sulfo-NHS-LC-biotin (Thermo Fisher Scientific, Waltham, MA) in PBS for 30 min on ice. The reaction was stopped by washing the cells with PBS containing 50 mM glycine. The surface biotinylated cells were lysed in 400 µl of RIPA buffer (50 mM Tris pH 8.0, 150 mM NaCl, 0.5% deoxycholate, 0.5% NP-40, 0.1% SDS, 1 mM Na_3_VO_4_, 1 µM microcystin-LR, and 1× proteinase inhibitor mix) and the biotinylated proteins were bound to immobilized NeutrAvidin agarose (Thermo Fisher Scientific). The bound proteins were washed and subjected to SDS-PAGE for immunoblotting. The absence of α-tubulin from the NeutrAvidin-bound fraction was used to confirm that the intracellular proteins were not biotinylated.

FLAG-tagged proteins were detected on the surface of live cells as follows. CAD cells were transfected with plasmids expressing the indicated FLAG-tagged proteins and cultured on poly-D-lysine-coated 8-well cover-glass chamber slides. The cells were washed with ice-cold PBS and incubated with 1 µg/ml anti-FLAG mouse monoclonal antibodies in 1% heat-inactivated goat serum/PBS at 4°C for 30 min. After being washed with ice-cold PBS three times, the cells were fixed with 4% PFA/PBS at 4°C for 30 min. The fixed cells were washed with PBS and further incubated with Cy3-conjugated donkey anti-mouse IgG in 1% heat-inactivated goat serum/PBS containing 5 µg/ml Hoechst 33342 (Invitrogen) to visualize the bound anti-FLAG antibodies and cell nuclei. The cells were washed with PBS, and their fluorescent images were serially collected with a BZ-9000 microscope (Keyence; Osaka, Japan) using the same conditions for each specimen. After collecting the images, the cells were incubated in 0.1% Triton X100/PBS for 5 min at room temperature. The resultant permeabilized cells were incubated with 1 µg/ml anti-FLAG mouse monoclonal antibodies in 1% heat-inactivated goat serum/PBS at 4°C for 30 min. After washing the cells with PBS, the bound anti-FLAG antibodies were visualized with the secondary antibodies. The resultant fluorescent images of the permeabilized cells were taken with the BZ-9000 microscope. Representative images of the same cells before and after permeabilization are shown in the figures.

### Analyses of alkaline phosphatase activity and p3-Alcα released into the cell culture medium

Plasmids expressing AP or the indicated AP-fusion proteins were transferred into CAD cells plated on a 12-well dish. Twenty-four hours later, the cells were washed with warmed fresh medium 3 times, and then 1 ml of medium was added to the cells. Ten-microliter culture medium was collected at the indicated time points. After finishing collection of the medium, the cells were washed and lysed with 400 µl of RIPA buffer (50 mM Tris pH 8.0, 150 mM NaCl, 0.5% deoxycholate, 0.5% NP-40, 0.1% SDS, 1 mM Na_3_VO_4_, 1 µM microcystin-LR, and 1× proteinase inhibitor mix). The AP activity of the collected medium and cell lysates was measured as described [15]. The p3 Alcα peptide generated from Alcα or its WA mutant was analyzed as described [9].

## Results

### Alcα protein is efficiently cleaved

**Figure 1:**
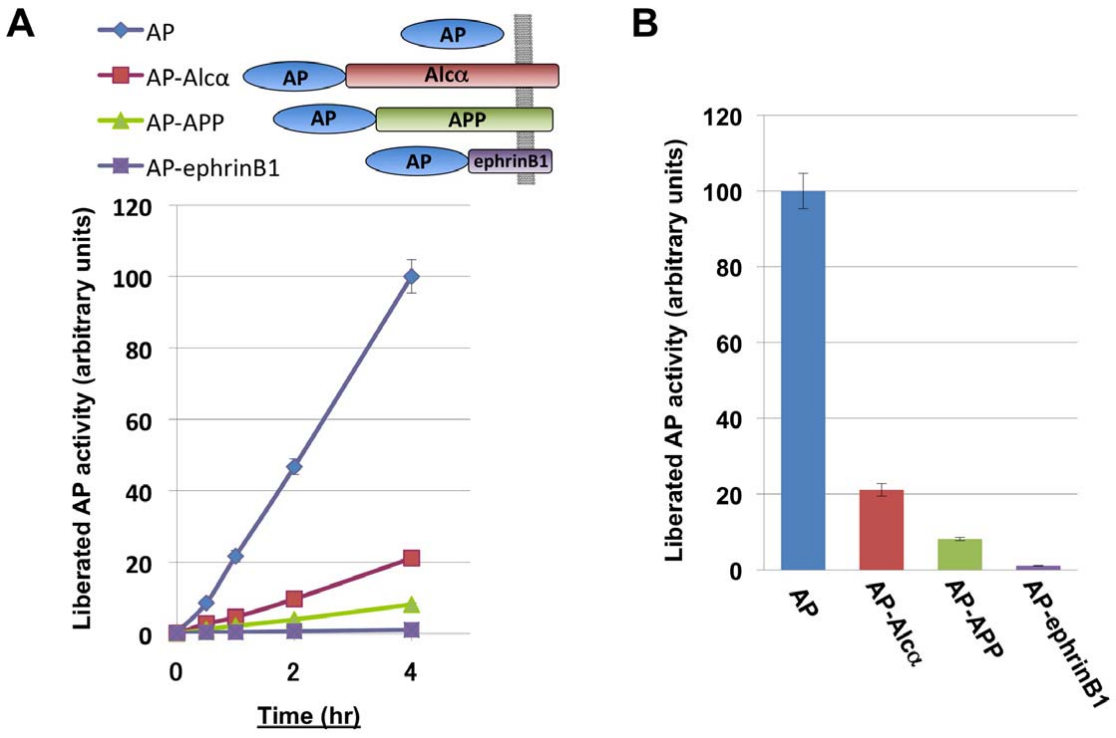
The Alcα N-terminal fragment is efficiently liberated into the extracellular space. A. One microgram of plasmid expressing secreted human placental alkaline phosphatase (AP) or its N-terminal fusion protein of Alcα, APP, or ephrinB1 was transfected into CAD cells (∼2×10^5^ cells). Twenty-four hours later, the cells were washed to exchange the culture medium. Ten microliters of the culture medium was collected at 30 min, 1 hr, 2 hrs, and 4 hrs after exchanging the medium, and the cells were collected and lysed in RIPA buffer. The AP activity in the culture medium was normalized to the activity in the corresponding cell lysate. The AP activity in the culture medium increased linearly over time. B. The released AP activity at the 4-hour time point. The amount of AP-Alcα liberated into the medium was ∼20% of that of freely secreted AP.

To quantify the extracellular secretion of the Alcα ectodomain, we prepared a plasmid expressing a fusion protein of Alcα and human placental alkaline phosphatase (AP). For comparison, we also prepared plasmids expressing a secreted form of AP alone or fusion proteins of AP with β-amyloid precursor protein (APP) or ephrinB1 (Figure 1A). The secreted form of human placental AP is known to be quickly and efficiently secreted, and is used as a standard in numerous assay systems (one recent example; [16]). APP is a well-characterized single-pass type I transmembrane protein that undergoes constitutive cleavage by ADAM10 [17], and its AP fusion protein was previously shown to be cleaved and secreted similar to wild-type APP [18]. EphrinB1 is cleaved by matrix metalloproteinases when treated with phorbol-12-myristate-13-acetate [19].

Plasmids expressing secreted AP or the AP fusion proteins were transfected into CAD mouse neuroblastoma cells, and the amount of AP activity released into the culture medium was measured starting 24 hrs after transfection. The released AP activity increased linearly with time (Figure 1A). The amount of secreted AP or AP fusion protein in the culture medium was normalized to the AP activity in the corresponding whole-cell lysate. We set this ratio for AP alone at the 4-hour time point to 100 (±4.7; S.E.M., n = 6), because secreted AP was the most efficiently liberated protein in this assay. Relative to AP, this value for the AP-APP fusion protein was 8.2±0.5 (n = 6); and for the AP-ephrinB1 fusion protein was 1.1±0.07 (n = 6). On the other hand, the value for the AP-Alcα fusion protein was surprisingly high, 21.1±1.6 (n = 6), indicating that the ectodomain of Alcα is cleaved and secreted very efficiently (Figure 1B).

### Alcα protein is rarely detected on the cell surface despite its transport to the periphery

**Figure 2:**
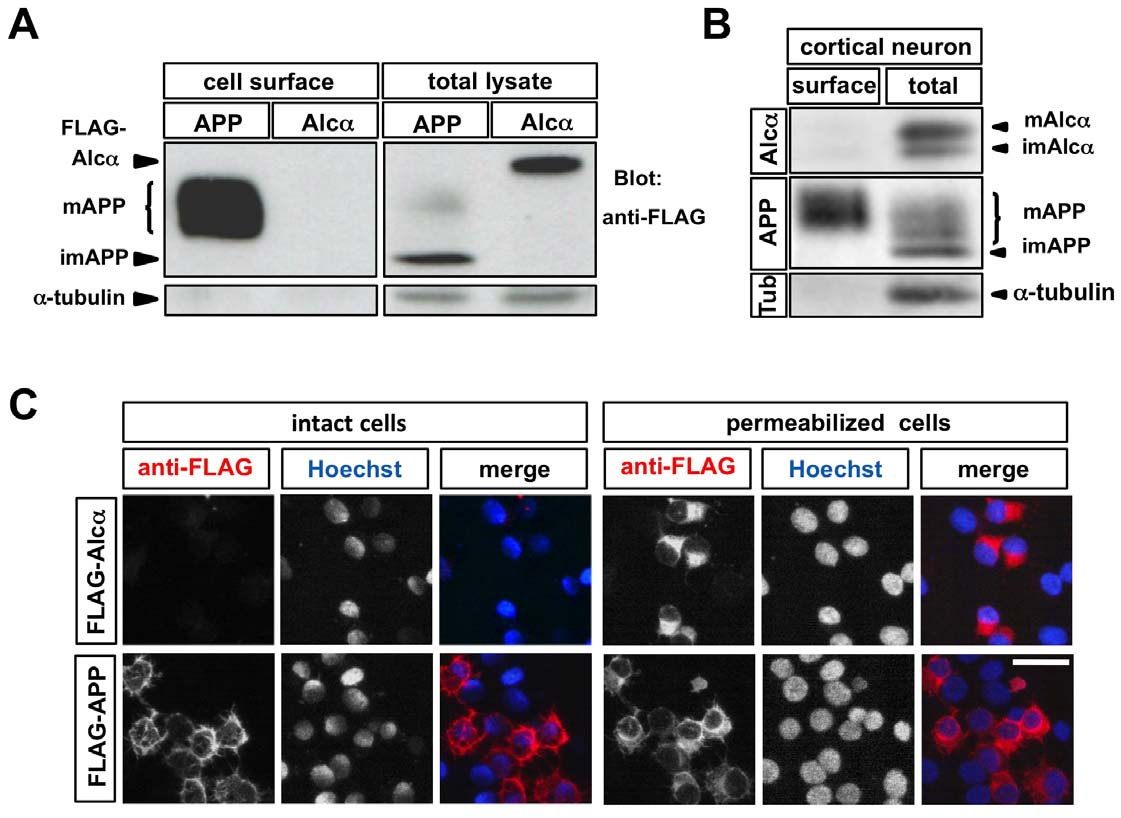
Full-length Alcα protein is rare on the cell surface. A. Surface biotinylation assay of CAD cells expressing APP or Alcα. One microgram of plasmid expressing FLAG-tagged APP (pcDNA3.1-FLAG-hAPP) or FLAG-tagged Alcα (pcDNA3.1-FLAG-hAlcα1) was transfected into CAD cells (∼2×10^5^ cells). Twenty-four hours later, the cells were incubated with Sulfo-NHS-LC-Biotin on ice. After quenching and washing, the cells were lysed in RIPA buffer, and part of the resultant lysate was incubated with NeutrAvidin agarose. The lysate (total lysate) and the NeutrAvidin agarose-bound fraction (cell surface) were subjected to SDS-PAGE and immunoblotting. mAPP: mature APP protein that already underwent both *N-* and *O-*glycosylation. imAPP: immature APP protein that was not *O-*glycosylated yet. Biotinylated cell-surface mAPP protein was concentrated in the NeutrAvidin agarose-bound fraction, while Alcα protein was rare in this fraction. B. Surface biotinylation assay of primary cultured mouse embryonic cortical neurons. Cortical neurons were isolated from E17.5 mouse brain, cultured for 5 days, and subjected to the cell-surface biotinylation assay as described above. The cell lysate (total) and NeutrAvidin agarose-bound fraction (surface) were subjected to SDS-PAGE and immunoblotting. mAlcα: mature Alcα protein that already underwent both *N-* and *O-*glycosylation. imAlcα: immature Alcα protein that was not *O-*glycosylated yet. Tub: α-tubulin. Alcα protein was rare in the NeutrAvidin agarose-bound fraction. C. Immunostaining of live intact CAD cells expressing APP or Alcα. CAD cells cultured on poly-D-lysine-coated cover-glass chamber slides were transfected with plasmids expressing FLAG-APP or FLAG-Alcα as described above. Twenty-four hours later, the cells were incubated with anti-FLAG antibodies for 30 min on ice. After the cells were washed and fixed, the bound anti-FLAG antibodies and cell nuclei were visualized by Cy3-conjugated anti-mouse IgG and Hoechst 33342, respectively. After capturing their images (intact cells), the cells were permeabilized with 1% Triton X100 in PBS at room temperature for 5 min, and incubated with anti-FLAG antibodies for 30 min on ice (permeabilized cells). After the cells were washed, the bound anti-FLAG antibodies and cell nuclei were visualized by Cy3-conjugated anti-mouse IgG and Hoechst 33342, respectively. Alcα protein was rarely observed on the surface of the cells that expressed Alcα, while APP protein was readily detected on surface of cells that expressed APP. Scale bar: 40 µm.

Because Alcα was cleaved to liberate its ectodomain at such high efficiency, we next examined whether the Alcα protein actually resides on the cell surface. To assess the amount of Alcα protein on the cell surface, we performed a surface biotinylation assay. N-terminal FLAG-tagged Alcα protein was expressed in CAD cells. For comparison, constitutively cleaved FLAG-tagged APP protein was expressed in parallel. To equalize the amount of expressed FLAG-tagged proteins in the cells, the ratio of the transfected expression plasmid to vacant vector was adjusted in advance. The surface proteins were biotinylated by incubating the cells with Sulfo-NHS-LC-Biotin at 4°C, and the biotinylated proteins were then bound to NeutrAvidin agarose. The glycosylated mature (*N*- and *O*-glycosylated) APP was specifically biotinylated and was recovered in the NeutrAvidin agarose-bound fraction, while neither immature (not yet *O*-glycosylated) APP nor cytoplasmic α-tubulin was detected in this fraction, indicating that the surface biotinylation assay was properly conducted (Figure 2A). In contrast, no biotinylated Alcα was detected under the same conditions (Figure 2A), suggesting that full-length Alcα protein was rare on the cell surface.

To examine whether endogenous Alcα resides on the cell surface, we performed the same surface biotinylation assay using cultured mouse cortical neurons. These neurons endogenously expressed APP and Alcα, as shown in Figure 2B. As anticipated, mature APP was readily biotinylated; however, mature Alcα was rarely biotinylated (Figure 2B), indicating that the endogenous Alcα protein was also rare on the cell surface of the primary cultured neurons.

To confirm these observations, live and intact CAD cells expressing the FLAG-tagged APP or FLAG-tagged Alcα protein under the same conditions were incubated with anti-FLAG antibodies at 4°C. After washing out the unbound antibodies, the cells were fixed, and the bound antibodies were visualized. The N-terminal FLAG-tagged APP protein was readily detected on the cell surface, whereas signals were rarely detected on the cells expressing FLAG-tagged Alcα (Figure 2C). The expressed Alcα protein could be detected with the anti-FLAG antibodies after the same cells were permeabilized with Triton X-100 (Figure 2C), confirming that full-length Alcα rarely resides on the cell surface.

**Figure 3:**
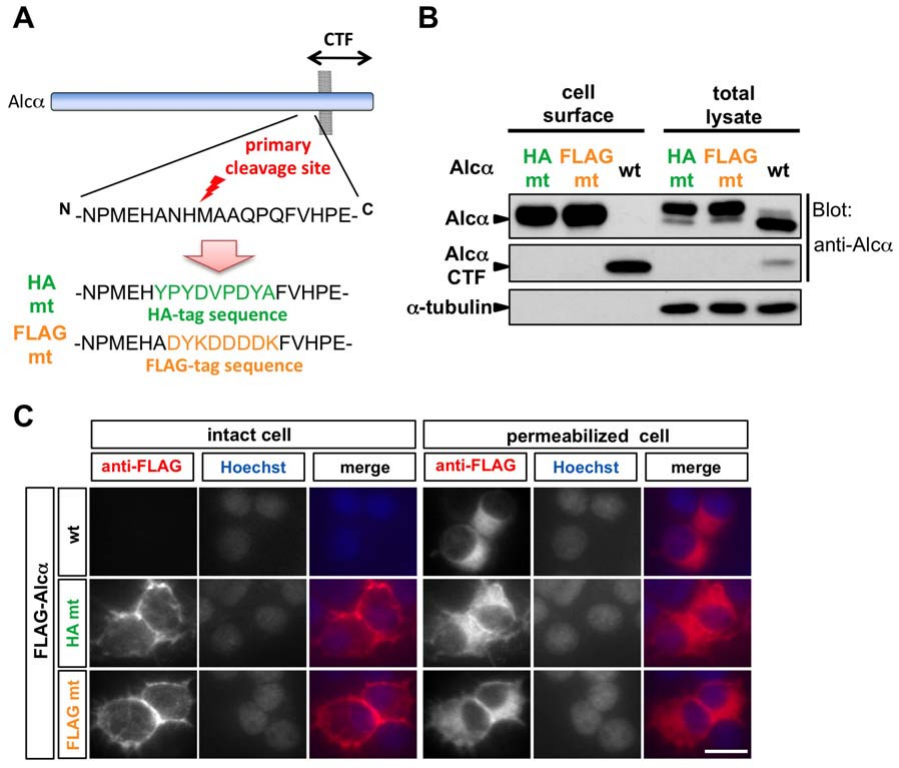
Uncleavable Alcα mutant proteins are transported to the cell surface. A. Uncleavable Alcα mutant proteins. The sequence around the primary cleavage site of human Alcα was replaced with that of the HA (HA mt) or FLAG (FLAG mt) tag. B. Surface biotinylation assay of CAD cells expressing wild-type or uncleavable mutant Alcα proteins. One microgram of plasmid expressing HA-tag-substituted (HA mt; pcDNA3.1-HAmt-hAlcα1), FLAG-tag-substituted (FLAG mt; pcDNA3.1-FLAGmt-hAlcα1), or wild-type (wt; pcDNA3.1-hAlcα1) Alcα was transferred into CAD cells, then a surface biotinylation assay was performed as in Figure 1A, and analyzed by immunoblotting using an anti-Alcα antibody (UT83) raised against the C-terminal cytoplasmic region. No C-terminal fragment (CTF) generated by primary cleavage was detected in the lysate of cells expressing the mutant proteins. Most of the mutant proteins were in the *N-* and *O-*glycosylated mature form and were readily detected on the cell surface. C. Immunostaining of live intact CAD cells expressing wild-type or uncleavable mutant Alcα proteins. CAD cells cultured on poly-D-lysine-coated cover-glass chambered slides were transfected with plasmids expressing FLAG-tagged wild-type (pcDNA3.1-FLAG-hAlcα1) or cleavage site substitution mutant Alcα (pcDNA3.1-HAmt-FLAG-hAlcα1 or pcDNA3.1-FLAGmt-FLAG-hAlcα1), and analyzed as in Figure 2C. The mutant Alcα proteins were readily observed on the cell surface. Scale bar: 20 µm.

One possible explanation for these observations is that Alcα is immediately endocytosed and/or rarely translocated to the plasma membrane. Alternatively, the ectodomain of Alcα may be cleaved off before or upon Alcα's reaching the plasma membrane, so virtually no full-length Alcα protein is detected on the cell surface. To examine these possibilities, we generated ‘uncleavable’ Alcα by replacing 8 or 9 amino acids around its primary cleavage site [9] with the HA- or FLAG-tag sequence, respectively (Figure 3A). These substitutions greatly reduced the amount of C-terminal cleavage product (CTF), indicating that the ectodomain of the mutants was not cleaved off by proteinases, or at least not as efficiently as the wild-type Alcα (Figure 3B).

If Alcα was immediately endocytosed and/or never transported to the plasma membrane, these uncleavable Alcα mutants would also rarely reside on the cell surface. However, the mutant proteins were readily biotinylated in the surface biotinylation assay (Figure 3B) and detected on the surface of live cells with anti-FLAG antibodies (Figure 3C). In addition, the proportion of slower-migrating mature Alcα protein in the cells expressing these uncleavable mutants was much higher than in those expressing wild-type proteins (Figure 3B), probably because the uncleaved mature Alcα protein accumulated on the cell surface. These observations suggested that Alcα is indeed transported to the plasma membrane, and supported the idea that the ectodomain is cleaved when Alcα is en route to and/or reaches the cell surface.

### Alcα protein can be cleaved en route to the cell surface

The observed efficient ectodomain shedding of Alcα, together with the finding that full-length Alcα was rarely retained on the cell surface, raised the possibility that Alcα can be cleaved, at least in part, within the cells, en route to the cell surface. To examine this possibility, we generated a mutant Alcα whose transport to the cell surface is attenuated. As previously described, Alcα is a cargo of kinesin-1; Alcα binds directly to the TPR repeat region of KLC, through its small (∼10 amino acids) WD motif structure [7], [8]. The WD motif is necessary and sufficient for Alcα's activity as a cargo of kinesin-1, and replacing the tryptophan residue in the WD motif with alanine abolishes this activity [8]. Since Alcα contains two WD motifs in its cytoplasmic region, the replacement of the tryptophan residues in both motifs with alanine should abolish its activity as a cargo of kinesin-1, and thus attenuate its transportation to the cell surface.

**Figure 4:**
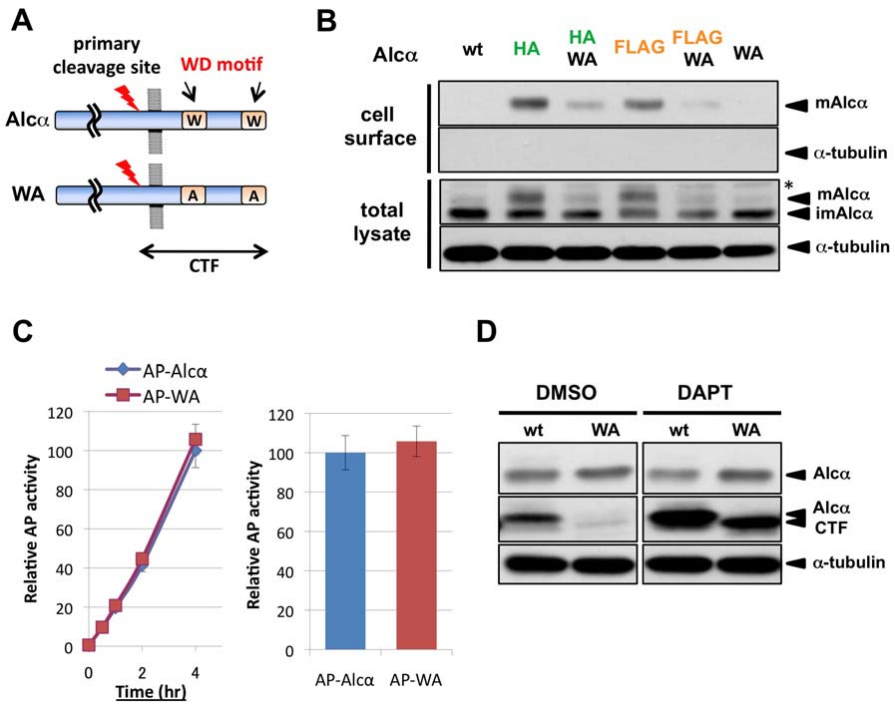
Attenuation of Alcα's peripheral transport does not affect its cleavage. A. Schematic representation of Alcα and its WA mutant. Alcα contains two WD motifs that associate with KLC1. Each WD motif has a tryptophan residue that is critical for its binding to KLC, and thus for Alcα's function as kinesin-1 cargo. Each tryptophan residue in the two WD motifs was replaced by alanine in the WA mutant Alcα. The WA mutation abolishes Alcα's KLC-binding activity [8]. B. Surface biotinylation assay of CAD cells expressing wild-type or mutant Alcα. Plasmids (0.5 µg) expressing uncleavable HA tag-substituted (pcDNA3.1-HAmt-hAlcα1), uncleavable FLAG tag-substituted (pcDNA3.1-FLAGmt-hAlcα1), wild-type (pcDNA3.1-hAlcα1) Alcα or their transport-defective WA mutant form (e.g., pcDNA3.1-HAmt-hAlcα1WA) were transferred into CAD cells (∼2×10^5^ cells). Twelve hours later, the cells were treated as in Figure 2B for the surface biotinylation assay. The amount of plasmid used for transfection and the incubation time after transfection were reduced to observe Alcα accumulation on the cell surface before reaching the saturation level; thus, the proportion of mature HA- or FLAG-substituted Alcα in the cell lysate was much smaller than in Figure 2B. The accumulation of HA- or FLAG-substitution ‘uncleavable’ mutant Alcα proteins on the cell surface was attenuated by the WA mutation. wt: wild-type Alcα, HA: HA tag-substitution uncleavable mutant Alcα, HA WA: HA tag-substitution uncleavable WA mutant Alcα, FLAG: FLAG tag-substitution uncleavable mutant Alcα, FLAG WA: FLAG tag-substitution uncleavable WA mutant Alcα, WA: WA mutant Alcα, *: non-specific signal appearing at this sensitivity. C. Liberation of the N-terminal fragment into the extracellular space by cells expressing wild-type or WA mutant Alcα. Plasmids (0.5 µg) expressing N-terminal AP-Alcα fusion protein (AP-Alcα) or its WA mutant (AP-WA) were transferred into CAD cells (∼2×10^5^ cells). Twelve hours later, the cells were washed to exchange the culture medium. A 10-µl aliquot of culture medium was collected at the indicated time points after exchanging the medium, and the cells were collected and lysed in RIPA buffer. The AP activity in the culture medium and cell lysate was measured, and the AP activity released into the culture medium over time was determined and compared as in Figure 3. No significant difference was observed in the amount of AP activity released from the cells expressing wild-type versus WA mutant AP-Alcα. D. Generation of the C-terminal fragment (CTF) of wild-type or WA mutant Alcα. Plasmid (0.5 µg) expressing wild-type (wt) or WA mutant (WA) Alcα was transferred into CAD cells (∼2×10^5^ cells). Twelve hours later, the γ-secretase inhibitor DAPT (1 µg/ml) or its solvent DMSO was added to the culture. Four hours after adding these reagents, the cells were collected, lysed in RIPA buffer, and subjected to immunoblotting. CTF from the WA mutant accumulated in the cells when its further cleavage by γ-secretase was inhibited by DAPT.

To verify that the tryptophan to alanine substitution of Alcα actually attenuates its transport to the cell surface, we expressed in CAD cells the HA- or FLAG-substituted uncleavable mutant Alcα in which the tryptophan residues of both WD motifs were replaced with alanines (WA mutant; Figure 4A), and examined their presence on the cell surface by the surface biotinylation assay. Since these uncleavable mutants readily accumulated on the cell surface, as shown in Figure 3B, we performed this assay 12 hrs after transfection, when these mutants were linearly accumulating on the cell surface. As anticipated, the WA mutation reduced the amount of surface-biotinylated uncleavable mutant Alcα proteins (Figure 4B). To visualize these proteins on the cell surface directly, live and intact CAD cells expressing the FLAG-tagged wild-type and mutant Alcα proteins under the same conditions were incubated with anti-FLAG antibodies at 4°C. The WA mutation greatly reduced the amount of cell-surface uncleavable Alcα protein (Figure S1). These observations indicated that the WA mutation attenuated the transport of Alcα protein to the plasma membrane.

We next quantified the amount of liberated ectodomain generated from the AP-fusion proteins of wild-type or WA mutant Alcα. Given that the transport of the WA mutant proteins to the cell surface was attenuated, if most of the Alcα protein was cleaved upon or after reaching the cell surface, the extracellular secretion of the cleaved ectodomain proteins should also be attenuated. However, no significant difference was observed in the amount of extracellularly liberated AP activity between the wild type- and WA mutant-expressing cells (Figure 4C). Collectively, these observations suggested that the Alcα protein is at least partly cleaved en route to the cell surface.

To examine if the cleavable WA mutant protein was properly processed, we next expressed the untransported mutant Alcα in CAD cells. The C-terminal fragment of the primary cleavage product (Alcα CTF) from the WA mutant was only faintly detected (Figure 4D), suggesting that the WA mutant might not be cleaved. However, the Alcα CTF is further cleaved by γ-secretase to produce p3-Alcα and Alcα ICD, and the latter is promptly degraded by an unspecified mechanism, so little Alcα ICD is detected in mouse brain, but Alcα CTF is readily detected [5]. To determine if Alcα CTF was actually produced from the WA mutant, the γ-secretase inhibitor DAPT was added to the culture. In the presence of DAPT, a comparable amount of Alcα CTF was produced from the WA mutant and wild-type Alcα (Figure 4D), indicating that the primary cleavage occurred in the WA mutant Alcα.

To further examine if another γ-secretase cleavage product, p3-Alcα peptide, was properly generated from the WA mutant, p3-Alcα peptide liberated into the culture medium was collected with anti-p3-Alc antibodies, and detected by mass spectrometry as described previously [9]. The p3-Alcα peptide was readily detected in the culture medium of the WA mutant-expressing cells as in that of wild-type Alcα expressing cells (Figure S2). The molecular weights of the p3-Alcα peptides (reflecting the cleavage sites) and their proportions derived from the WA mutant were identical to those derived from wild-type Alcα (Figure S2). These findings collectively indicated that the WA mutant Alcα was properly processed to generate *bona fide* p3-Alcα peptide, and thus that this processing was largely unaffected by the inappropriate retention of Alcα in the cells.

### Attenuation of Alcα cleavage leads to aberrant peripheral localizations of KLC1 and kinesin heavy chain (KIF5C)

We next addressed why Alcα, which is produced as a transmembrane protein, needs to be cleaved with such remarkable efficiency en route to the cell surface that little full-length Alcα protein resides there. As described previously, Alcα binds to KLC to activate kinesin-1's association with Alcα–containing vesicles [7], and Alcα's WD motif is sufficient to recruit kinesin-1 to these vesicles to activate their anterograde transport [8]. These findings led us to speculate that, if Alcα stayed on the cell surface, it might inappropriately recruit kinesin-1 to the cell periphery.

**Figure 5:**
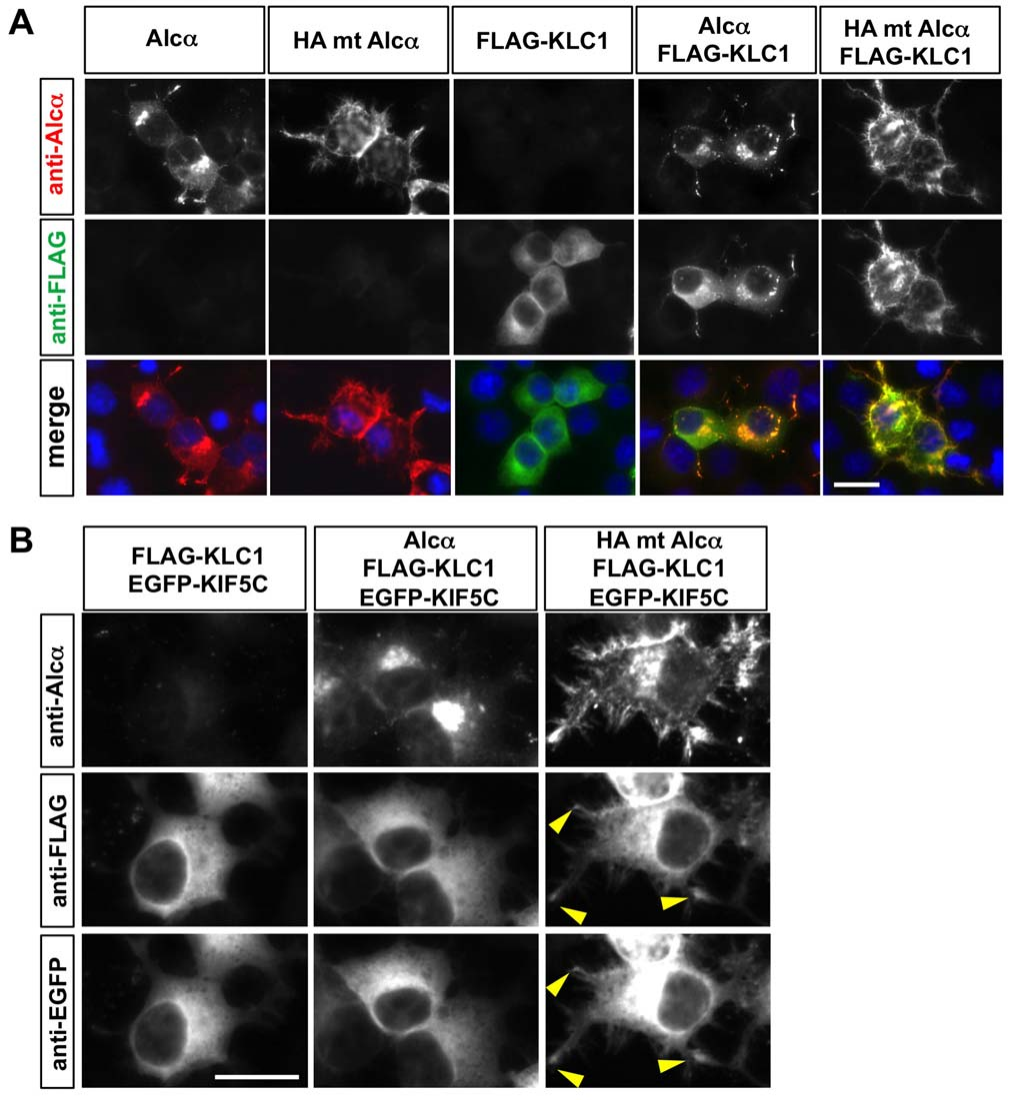
Uncleavable Alcα aberrantly recruits kinesin-1 to the cell periphery. A. Uncleavable Alcα leads improper peripheral retention of KLC1. CAD cells cultured on poly-D-lysine-coated cover-glass chamber slides were transfected with plasmid expressing wild-type Alcα (pcDNA3.1-hAlcα1), uncleavable HA tag-substituted Alcα (pcDNA3.1-HAmt-hAlcα1), and/or FLAG-tagged KLC1 (pcDNA3.1-FLAG-KLC1) as indicated. Twenty-four hours later, the cells were fixed, permeabilized, and incubated with anti-FLAG (M2) and anti-Alcα (UT83) antibodies. The bound antibodies were visualized with anti-mouse FITC-conjugated and anti-rabbit Cy3-conjugated antibodies. The blue signals in the merged pictures are nuclei stained with DAPI. The FLAG-KLC1 protein was aberrantly accumulated near the periphery in cells expressing the HA tag-substituted Alcα. Scale bar: 20 µm. B. KIF5C is partially retained at the tips of cellular protrusions in cells expressing uncleavable Alcα. CAD cells cultured on poly-D-lysine-coated cover-glass chamber slides were transfected with plasmids expressing FLAG-tagged KLC1 (pcDNA3.1-FLAG-KLC1), EGFP-KIF5C (pEGFP-KIF5C), and wild-type Alcα (pcDNA3.1-hAlcα1) or uncleavable HA tag-substituted Alcα (pcDNA3.1-HAmt-hAlcα1), as indicated. Twenty-four hours later, the cells were fixed, permeabilized, and incubated with anti-FLAG (M2), anti-Alcα (col90), and anti-GFP (598) antibodies. The bound antibodies were visualized with anti-mouse Cy5-conjugated, anti-guinea pig Cy3-conjugated, and anti-rabbit FITC-conjugated antibodies, respectively. FITC and Cy5 double-positive signals were frequently observed at the tips of cellular protrusions in cells expressing the HA tag-substituted uncleavable Alcα. Scale bar: 20 µm.

To assess the possibility, we expressed an uncleavable Alcα mutant protein together with KLC1 in CAD cells. The FLAG-tagged KLC1 protein expressed alone was evenly distributed in the cytoplasm (Figure 5A). When expressed with wild-type Alcα, the KLC1 protein was still distributed throughout the cytoplasm, and some was co-localized with Alcα, as previously reported ([7]; Figure 5A). However, when expressed with the uncleavable mutant Alcα, most of the expressed KLC1 protein was localized along the cell surface, where the uncleaved Alcα protein resided (Figure 5A). These findings indicated that the peripherally located Alcα could inappropriately recruit KLC1 near the cell surface.

To determine whether kinesin-1, which is composed of KLC and KIF5 (kinesin heavy chain, KHC), could also be recruited to the cell periphery when Alcα was retained in the plasma membrane, KLC1, KIF5C, and wild-type or uncleavable Alcα were co-expressed in CAD cells. Kinesin-1 protein was evenly distributed in the cytoplasm, and rarely accumulated at the cell periphery (Figure 5B). This distribution was not significantly altered by the presence of wild-type Alcα (Figure 5B). However, when expressed with uncleavable Alcα, the KIF5C and KLC1 proteins were detected in spiky cellular protrusions, in which they often accumulated at the tips (Figure 5B, some of these tips are indicated by arrowheads). The peripheral recruitment of KLC1 was greatly reduced by the co-expression of KIF5C. This might be partly because the kinesin-1 holoenzyme (KIF5C-KLC1 in this experiment) would be readily transported retrogradely when detached from the peripheral Alcα, with vesicles transported by cytoplasmic dynein motors [20]. Our observations collectively suggest that the aberrant peripheral accumulation of Alcα perturbs the appropriate intracellular distribution of kinesin-1.

**Figure 6:**
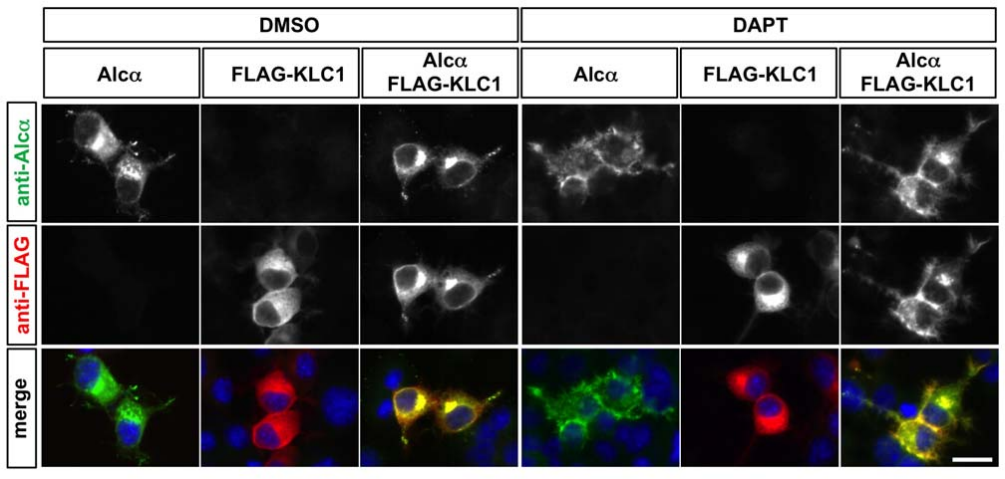
Alcα CTF accumulated along the cell surface leads improper peripheral retention of KLC1. CAD cells cultured on poly-D-lysine-coated cover-glass chamber slides were transfected with plasmid expressing wild-type Alcα (pcDNA3.1-hAlcα1) and/or FLAG-tagged KLC1 (pcDNA3.1-FLAG-KLC1) as indicated. Twenty-four hours later, the γ-secretase inhibitor DAPT (1 µg/ml) or its solvent DMSO was added to the culture. Four hours after adding these reagents, the cells were fixed, permeabilized, and incubated with anti-FLAG (M2) and anti-Alcα (UT83) antibodies. The bound antibodies were visualized with anti-mouse Cy3-conjugated and anti-rabbit FITC-conjugated antibodies. Addition of DAPT accumulated Alcα CTF proteins on the cell surface. Please note that the anti-Alcα antibody recognizes the C-terminal cytoplasmic domain of Alcα, and visualizes both full length Alcα and Alcα CTF. The blue signals in the merged pictures are nuclei stained with DAPI. The FLAG-KLC1 protein was aberrantly recruited to the periphery in the cells co-expressing Alcα and cultured with DAPT. Scale bar: 20 µm.

We have shown that Alcα CTF proteins reside on the cell surface (Figure 3B). Given that cytoplasmic domain of Alcα is sufficient for binding to KLC [7], accumulation of Alcα CTF in the plasma membrane may also inappropriately recruit KLC1 to the cell periphery. To assess the possibility, KLC1 and/or Alcα were expressed in CAD cells. 24 hrs after transfection, the γ-secretase inhibitor DAPT or its solvent DMSO was added to the culture. Addition of DMSO did not alter the distribution of Alcα and KLC1 in the cells. Four hours after adding DAPT, Alcα CTF proteins were accumulated on the cell surface (Figure 6). The FLAG-tagged KLC1 proteins expressed alone were distributed in the cytoplasm after addition of DAPT to the culture. However, when expressed with Alcα, majority of KLC1 proteins were localized along the cell surface, where the accumulated Alcα CTF proteins resided (Figure 6). These observations further confirmed that improper retention of the Alcα cytoplasmic domain around the cell surface leads to aberrant peripheral localization of KLC1.

## Discussion

Here we showed that the evolutionarily conserved type I transmembrane protein Alcα is constitutively cleaved, and the resultant soluble ectodomain (sAlcα) is efficiently liberated to the extracellular space; its release rate was ∼20% of that of a freely secreted protein. We found that this ectodomain shedding occurs, at least in part, en route to the plasma membrane, and little full-length Alcα protein could be detected on the cell surface. We further showed that uncleaved Alcα protein accumulated on the cell surface and induced the aberrant retention of KLC1 and KIF5C at the cell periphery.

Kinesin-1 plays a major role in fast axonal transport, in which a defect might contribute to the onset of neurodegenerative disorders (reviewed in [21]). An improper peripheral accumulation of kinesin-1 might decrease the amount of available kinesin-1 molecules in a cell, which could attenuate the axonal transport driven by kinesin-1. Alcα binds to KLC through WD motifs in its C-terminal cytoplasmic region; thus, the cytoplasmic region needs to be cleaved off from Alcα in the plasma membrane to prevent kinesin-1 from associating with Alcα. γ-secretase is the enzyme responsible for this cleavage [10], which requires the prior shedding of the extracellular domain. Evidence presented here indicates that the N-terminal extracellular domain of Alcα is cleaved off at least in part en route to the cell surface. This prior ectodomain shedding may facilitate the secondary cleavage by γ-secretase at Alcα's destination. The attenuation of these Alcα cleavages may result in inappropriate accumulation of full length Alcα and/or Alcα CTF proteins around the cell surface, which could lead aberrant peripheral retention of kinesin-1. Further precise analyses will be required to evaluate the functional relevance of the observed constitutive cleavage of Alcα in the general axonal transport system in neurons.

It should be noted that Alcα is not merely a passive cargo of kinesin-1. Kinesin-1 adopts a folded, inactive form in its quiescent state [22]. Alcα can initiate the activation of kinesin-1 without requiring the expression of additional factors in the cell [8]. This feature of Alcα is unusual; another well-characterized kinesin-1 cargo protein JIP-1 requires the additional expression of FEZ-1 to activate kinesin-1 [23], and JIP-1 cannot activate the anterograde transport of JIP-1-containing vesicles by itself [8]. Peripherally retained Alcα might not only recruit kinesin-1 but also inappropriately activate it, resulting in undesirable effects. This might be one of the reasons why Alcα needs to be constitutively cleaved with such high efficiency. Further investigation is necessary to elucidate what kind of unfavorable events would be caused by the aberrant peripheral localizations of Alcα and kinesin-1.

The function of Alcα's cleaved N-terminal extracellular domain is presently unknown. It contains two cadherin-like repeat structures, and like the rest of the molecule, is evolutionarily conserved (32% identity, 52% similarity between *C. elegan*s and humans). CASY-1, an ortholog of Alcadein in *C. elegan*s, was shown to be required for salt chemotaxis learning [24], [25], and its soluble N-terminal ectodomain can rescue the learning defects caused by mutations of casy-1 [24], indicating that the secreted N-terminal fragment plays an important role in this process. In the case of APP, which is constitutively cleaved by ADAM10 [17], the ectodomain can rescue the APP-deficient phenotype in mice [26], indicating that the ectodomain is a functional product. This interpretation is supported by other *in vitro* and *in vivo* studies (reviewed by [27]). Further analysis of Alcα cleavage products may shed light on the functionality of its cleavage products and those of other constitutively cleaved type I transmembrane proteins.

Cellular and tissue homeostasis is maintained by the exquisite functional combination of a wide variety of cellular factors. The observed constitutive cleavage of the kinesin-1 cargo protein Alcα may play a role in maintaining cellular homeostasis, in part by preventing its vehicle, kinesin-1, from being retained at its destination at the cell surface. Further investigation of this unique membrane protein may provide insight into such mechanisms, whose failure might become a cause of neurodegenerative diseases.

## Supporting Information

Figure S1 **The peripheral accumulation of uncleavable Alcα is attenuated by the WA mutation.** CAD cells cultured on poly-D-lysine-coated cover-glass chamber slides were transfected with plasmids expressing FLAG-Alcα wild-type (pcDNA3.1-FLAG-hAlcα1; wt) or mutant proteins (pcDNA3.1-HAmt-FLAG-hAlcα1; HA mt, pcDNA3.1-FLAGmt-FLAG-hAlcα1WA; HA WA, pcDNA3.1-FLAGmt-FLAG-hAlcα1; FLAG mt, pcDNA3.1-FLAGmt-FLAG-hAlcα1WA; FLAG WA, or pcDNA3.1-FLAG-hAlcα1WA; WA) as in Figure 4B and analyzed as in Figure 1C and 3C. The W to A mutation of both WD motifs reduced the accumulation of uncleavable Alcα protein on the cell surface. Scale bar: 20 µm.
(TIF)

Figure S2 **The p3-Alc peptide generated from the WA mutant Alcα protein was identical to that generated from wild-type Alcα protein.** A. The p3-Alc peptides generated from Alcα proteins. The single primary cleavage site and multiple secondary cleavage sites generated various species of p3-Alc peptides, named as shown. B. The WA mutation did not alter the cleavage sites or the preference of secondary cleavage sites of Alcα. The p3-Alc peptides generated from HEK293 cells transfected with plasmids expressing Alcα (pcDNA3.1-hAlcα1) or its WA mutant (pcDNA3.1-hAlcα1WA) were collected and subjected to MALDI-TOF-MS analysis as described in [9]. The MS spectra of the p3-Alc peptides collected from these cells were essentially identical, suggesting that the WA mutant protein was cleaved in the same manner as the wild-type Alcα protein.
(TIF)
