# Supplementary figures and images for: Constitutive Cleavage of the Single-Pass Transmembrane Protein Alcadeinα Prevents Aberrant Peripheral Retention of Kinesin-1
Source: PLoS One. 2012 Aug 8;7(8):e43058. doi: 10.1371/journal.pone.0043058 (PMC3414480; doi:10.1371/journal.pone.0043058)

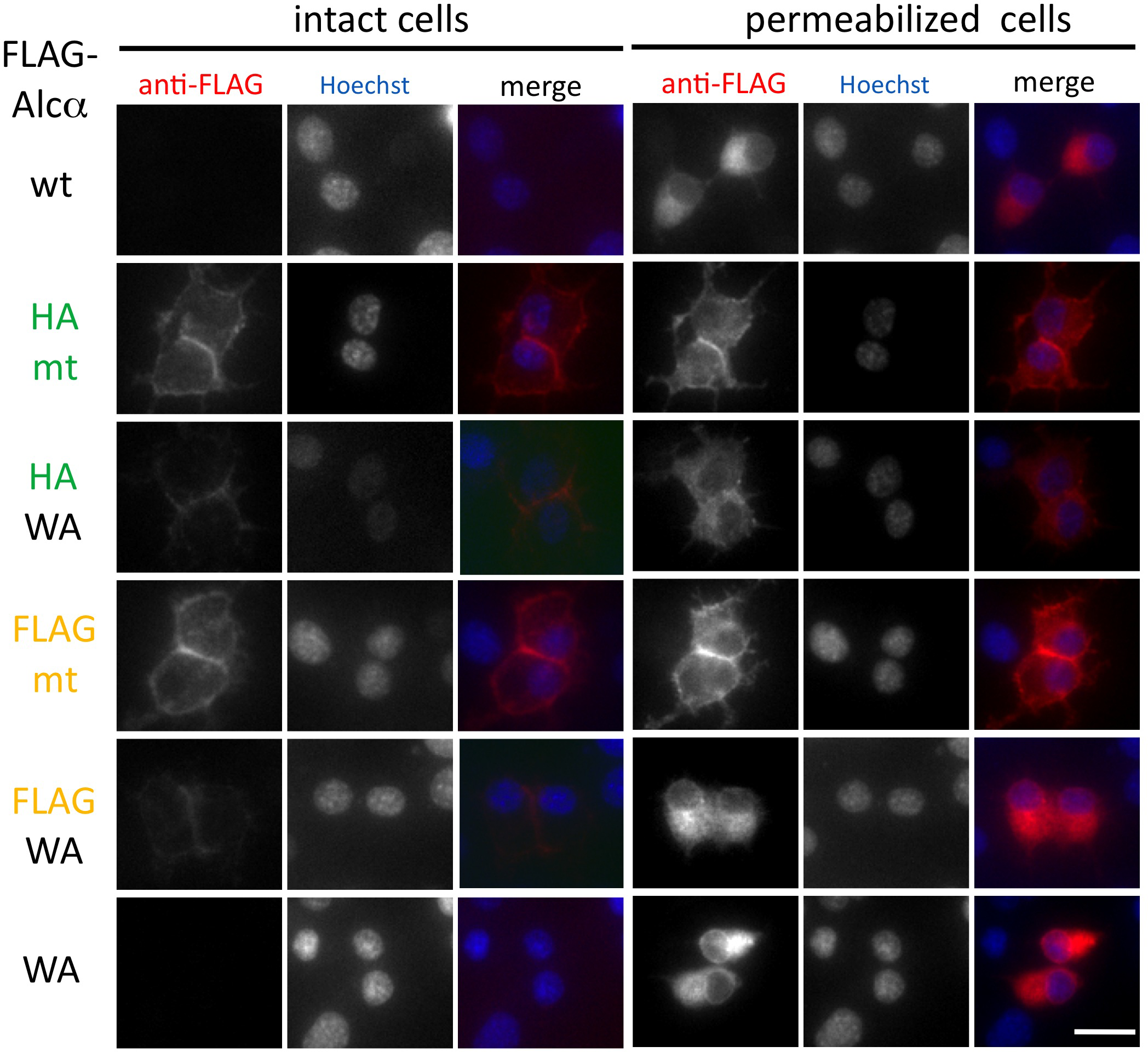

Supplement: Figure S1 — The peripheral accumulation of uncleavable Alcα is attenuated by the WA mutation. CAD cells cultured on poly-D-lysine-coated cover-glass chamber slides were transfected with plasmids expressing FLAG-Alcα wild-type (pcDNA3.1-FLAG-hAlcα1; wt) or mutant proteins (pcDNA3.1-HAmt-FLAG-hAlcα1; HA mt, pcDNA3.1-FLAGmt-FLAG-hAlcα1WA; HA WA, pcDNA3.1-FLAGmt-FLAG-hAlcα1; FLAG mt, pcDNA3.1-FLAGmt-FLAG-hAlcα1WA; FLAG WA, or pcDNA3.1-FLAG-hAlcα1WA; WA) as in Figure 4B and analyzed as in Figure 1C and 3C. The W to A mutation of both WD motifs reduced the accumulation of uncleavable Alcα protein on the cell surface. Scale bar: 20 µm. (TIF) [file pone.0043058.s001.tif]

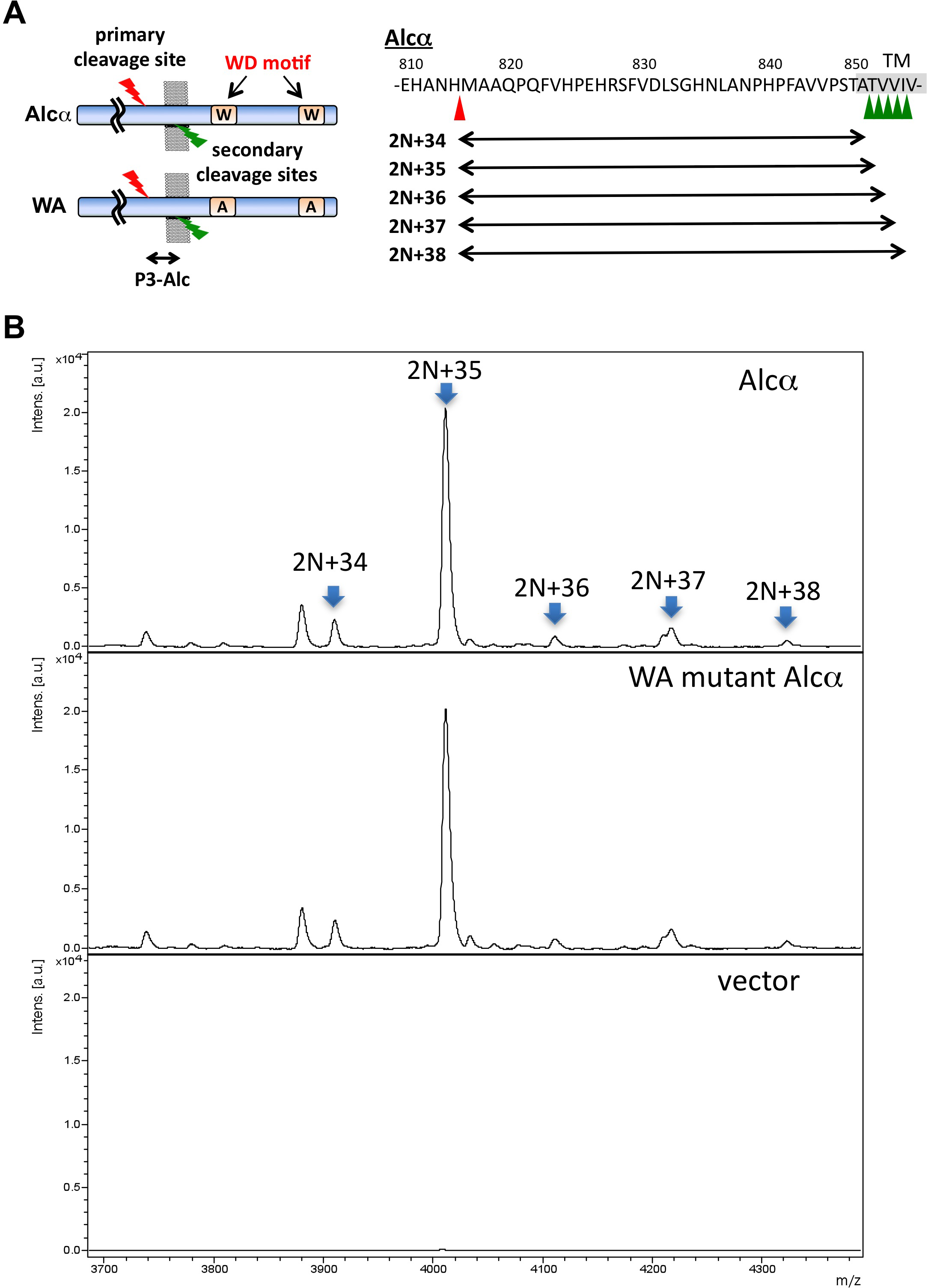

Supplement: Figure S2 — The p3-Alc peptide generated from the WA mutant Alcα protein was identical to that generated from wild-type Alcα protein. A. The p3-Alc peptides generated from Alcα proteins. The single primary cleavage site and multiple secondary cleavage sites generated various species of p3-Alc peptides, named as shown. B. The WA mutation did not alter the cleavage sites or the preference of secondary cleavage sites of Alcα. The p3-Alc peptides generated from HEK293 cells transfected with plasmids expressing Alcα (pcDNA3.1-hAlcα1) or its WA mutant (pcDNA3.1-hAlcα1WA) were collected and subjected to MALDI-TOF-MS analysis as described in [9]. The MS spectra of the p3-Alc peptides collected from these cells were essentially identical, suggesting that the WA mutant protein was cleaved in the same manner as the wild-type Alcα protein. (TIF) [file pone.0043058.s002.tif]
